# T lymphocytes isolated from patients with advanced colorectal cancer are suitable for gene immunotherapy approaches

**DOI:** 10.1038/sj.bjc.6600857

**Published:** 2003-04-01

**Authors:** A J Sheen, D J Sherlock, J Irlam, R E Hawkins, D E Gilham

**Affiliations:** 1 Cancer Research UK Department of Medical Oncology, Paterson Institute for Cancer Research, Christie Hospital NHS Trust, University of Manchester, Wilmslow Road, Manchester M20 4BX, UK; 2 Department of Surgery, North Manchester Healthcare NHS Trust, Manchester M8 5RB, UK

**Keywords:** T lymphocyte, patient, tumour;, retrovirus, chimeric, receptor

## Abstract

Despite improvements in treatment, the 5-year survival for metastatic colorectal cancer remains poor. Novel approaches such as gene immunotherapy are being investigated to improve treatment. Retroviral gene transfer methods have been shown to transduce primary human T lymphocytes effectively resulting in the expression of therapeutic genes. However, a number of defects have been identified in T lymphocytes isolated from patients bearing tumour, which may have critical implications for the development of gene-targeted T cells as an anticancer therapy. To address this issue, primary T lymphocytes were isolated from patients with advanced colorectal cancer and tested for their ability to be transduced and to express subsequently a chimeric immune receptor consisting of a single-chain antibody fragment antigen-binding moiety specific for carcinoembryonic antigen (CEA) fused to the T cell receptor (TCR) CD3*ζ* chain. In 10 out of 10 patients, T lymphocytes were transduced, expanded in the absence of selection and tested for functional activity against CEA-expressing tumour cells. In each case, functional-specific cytotoxic activity was observed. Negligible activity was found in control cultures. This study highlights the feasibility of patient-derived T lymphocytes as a source of immune cells for autologous gene immunotherapy approaches.

Colorectal cancer affects approximately 300 000 people in Europe and the US each year (Midgley and Kerr, 1999), accounting for 20–30 000 deaths in the UK alone (Curley and Vecchio, 1998). Surgery can cure selected patients with liver metastases (DeMatteo *et al*, 2000; Biasco and Gallerani, 2001). However, despite continued advances in systemic treatment, the overall 5-year survival has not improved significantly for advanced colorectal disease (Midgley and Kerr, 1999).

Our aim is to utilise the power of the immune system as a novel form of cancer therapy. Gene therapy techniques have been developed to modify T lymphocytes in order to target and lyse colorectal tumour cells through the development of chimeric immune receptors (CIRs) (Hombach *et al*, 1999; Nolan *et al*, 1999; Beecham *et al*, 2000; Daly *et al*, 2000). CIRs are composed of antigen binding domains, usually derived from single-chain antibody fragments (scFv) (Hawkins *et al*, 1998), fused to the cytosolic domains of important signalling receptors (Gross *et al*, 1989) (Figure 1A

**Figure 1:**
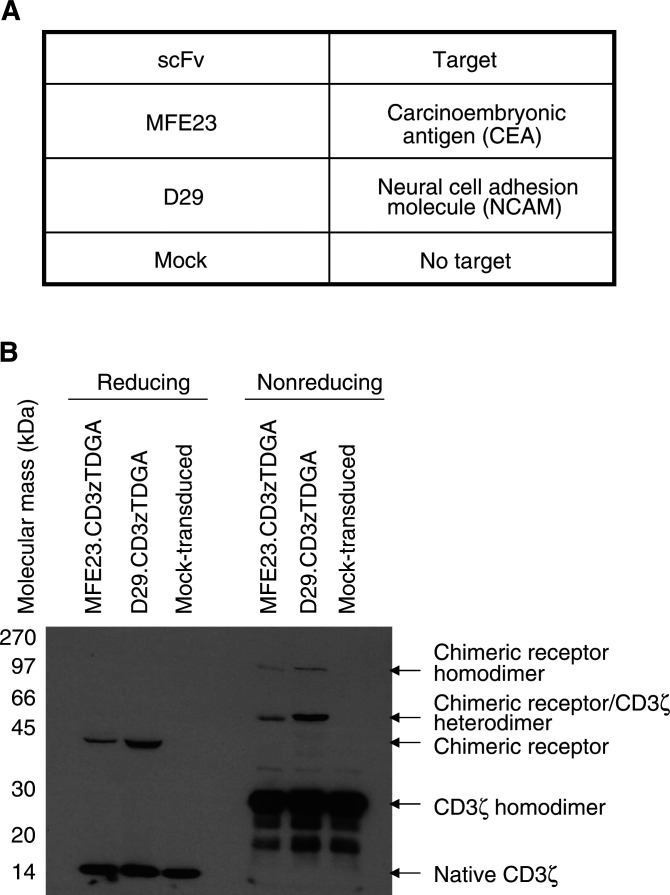
Expression of chimeric receptor proteins in primary human T lymphocytes derived from patients with advanced colorectal cancer. (**A**) The specificity of the scFv used in these studies. (**B**) Chimeric receptor transduced and mock patient T lymphocytes, at time of cytotoxicity assays, were lysed with RIPA and subjected to reducing or nonreducing SDS–PAGE and transferred to nitrocellulose filters. Blots were probed with anti-CD3*ζ* Mab (1 : 1000 dilution, Pharmingen, Oxford, UK) with a secondary antibody of anti-mouse Fc-HRP conjugate (1 : 2000) and immunoreactive bands visualised using ECL™ (Amersham, UK). Under reducing conditions, the endogenous CD3*ζ* protein was identified as a 16 kDa band in all samples with the chimeric receptors clearly identified as single immunoreactive bands at the predicted molecular mass of 43 kDa. Chimeric homodimers (86 kDa) and heterodimers (60 kDa) formed with the endogenous CD3*ζ* were identified along with CD3*ζ* homodimers (32 kDa) under nonreducing conditions.

). Our present work has focused upon the targeting of carcinoembryonic antigen (CEA) through a CEA-specific scFv fused to the CD3*ζ* chain. CD3*ζ* is thought to play the predominant role in T-cell signalling resulting in T-cell effector activity (Haynes *et al*, 2001). Binding of CEA by the CEA-specific CIR generates downstream activation signals resulting in cytokine production and target cell cytotoxicity in the absence of the usual restrictive mechanisms required for T-cell activation (Hombach *et al*, 1999; Nolan *et al*, 1999; Beecham *et al*, 2000; Daly *et al*, 2000). This approach results in the generation of large numbers of antigen-specific T cells. Importantly, since the CIRs recognise whole-protein antigens, tumour cells should not be able to avoid immune detection by the downregulation of immunoregulatory molecules such as major histocompatibility (MHC) molecules.

The CIRs used in this study were either an anti-CEA-specific scFv, MFE23 (derived by phage display technology) (Chester *et al*, 1994; Begent *et al*, 1996) or a control scFv (anti-neural cell adhesion molecule (NCAM)) (D29) (Whittington *et al*, 2001) fused to the human CD3*ζ* protein. Both receptors have been shown to be active when introduced into primary human T lymphocytes derived from healthy donors (Gilham *et al*, 2002). Importantly, in T cells derived from these normal, healthy donors, the presence of soluble CEA did not inhibit the cytolytic activity of the gene-modified T lymphocytes suggesting that high-serum CEA should not interfere with the activity of the anti-CEA CIR in patients.

However, important questions remain concerning the viability of this approach for clinical use. A number of studies have demonstrated that T lymphocytes isolated from tumour-bearing mice (Mizoguchi *et al*, 1992) and patients (Bukowski *et al*, 1998) are dysfunctional owing to altered expression of key proteins including members of the CD3 complex. T lymphocytes isolated from patients with advanced melanoma (Saito *et al*, 2000), and head and neck cancer (Hoffman *et al*, 2002) have also been shown to be more susceptible to spontaneous apoptosis thereby potentially compromising the patient's own immune competence. Furthermore, tumour-infiltrating lymphocytes isolated from colorectal hepatic metastases have been shown to have reduced CD3*ζ* chain expression and, upon expansion, lack tumour-specific lytic activity (Yoong and Adams, 1998). In view of this work and to further determine the viability of our approach in a clinical setting, T lymphocytes were isolated from the peripheral blood of patients with advanced colorectal disease (hepatic colorectal metastases) in order to determine whether they could be efficiently gene modified to express a chimeric immune receptor. Subsequently, the functional activity of the CIR in T lymphocytes was assessed through its ability to induce cytolytic activity when challenged with tumour cell lines *in vitro.*

## MATERIALS AND METHODS

### Reagents

All chemical reagents except where stated were purchased from Sigma (Dorset, UK). Anti-CD3*ɛ* (OKT3) was purchased from Orthobiotech (USA) and anti-hCD28 from R&D Systems (Minneapolis, MN, USA). Chiron (Amsterdam, The Netherlands) supplied the recombinant human IL-2.

### Cell culture

All cell culture materials were obtained from Life Sciences (Paisley, Scotland). The human CEA-positive cell lines MKN45 K (human gastric carcinoma, kindly provided by Dr J Embleton, PICR), LS174T (human colon adenocarcinoma, ECACC No. 87060401), LoVo (human colon adenocarcinoma, ECACC No. 87060101), NCAM positive SK-N-BE (human neuroblastoma, ATCC No. CCRL-2271), PG13 retroviral producer cells (Miller *et al*, 1991) and HeLa (human cervix epithelial carcinoma, ECACC No. 93021013) cell lines were all cultured in Dulbecco's modified Eagle's medium (DMEM) with 10% foetal bovine serum (FBS, Life Technologies, Paisley, Scotland). Patient T lymphocytes were cultured in T-cell media (RPMI 1640, 25 mM HEPES, 2 mM glutamine and 5 × 10^5^ 2-mercaptoethanol with the addition of 10% FBS). All cells were maintained in a 37°C 5% CO_2_/air incubator.

### Production of recombinant receptors encoding CIRs

Stable PG13 cell lines producing either rkat.MFE23.CD3*ζ*TDGA.IRES.EGFP and rkat.D29.CD3*ζ*TDGA.IRES.EGFP recombinant retroviruses have been previously described (Gilham *et al*, 2002). These vectors consist of the MFE23 scFv (specific for CEA) or the D29 scFv (specific for NCAM) fused to the CD3*ζ* receptor protein with the enhanced green fluorescent protein (EGFP) coexpressed by means of an internal ribosome entry site (IRES) element. Cell culture media were aspirated from PG13 cell monolayers (approximately 70–80% confluent) and replaced with T-cell media. Retrovirus was collected after an overnight incubation and then filtered through a 0.45 *μ*m filter prior to its use in transduction. T-cell media were replaced onto the PG13 monolayer with more virus collected and filtered after an overnight incubation for second and subsequent transductions.

### Patient details

All patients selected were due to undergo hepatic resection for colorectal liver metastases and were consented for the purposes of this study as approved by North and South Manchester Ethics committees. The median age of the patients was 66 years (range 42–73 years, mean 65.2±9.9 years). No patient had received any form of chemotherapy for at least 1 month prior to T lymphocyte harvest. Preoperative serum CEA levels and operations performed are listed in Table 1

**Table 1:** A summary of patient details, including operation and preoperative serum CEA levels, with T lymphocyte population phenotypes after retroviral transduction and expansion

**Patient (age in years)**	**ScFv fused to CD3ζTDGA**	**Preoperative CEA (*μ*g l^−1^)**	**Operation**	**% CD3+**	**% CD3+, GFP+**	**% CD8+, GFP+**	**% CD4+, GFP+**	**Ratio of transduced CD8+ : CD4+**	**Ratio of total CD8+ : CD4+ T cells**
1 (70)	Mock	35	Left hepatectomy	99	0	0	0	—	11.5 : 1
	MFE23			99	20	12	3	4 : 1	8.9 : 1
	D29			99	15	17	3	5.7 : 1	9.1 : 1
2 (66)	Mock	6	Right hepatectomy	99	0	0	0	—	4.2 : 1
	MFE23			99	5	5	2	2.5 : 1	4.9 : 1
	D29			99	7.5	7	1	7 : 1	4.5 : 1
3 (64)	Mock	1	Right hepatectomy	96	1	0	0	—	2.3 : 1
	MFE23			91	21	19	16	1.2 : 1	1.5 : 1
	D29			90	13	9	6	1.2 : 1	1.5 : 1
4 (70)	Mock	9	Right hepatectomy	99	0	0	0	—	1.9 : 1
	MFE23			94	21	19	8	2.4 : 1	2.1 : 1
	D29			97	9	11	5	2.2 : 1	2.2 : 1
5 (42)	Mock	155	Left hepatectomy+segmentectomy	88	1	2	1	2 : 1	1.3 : 1
	MFE23			93	33	17	13	1.0 : 1	1.4 : 1
	D29			89	23	20	14	1.4 : 1	1.4 : 1
6 (73)	Mock	>1500	Right Hepatectomy	89	0	3	0	—	10 : 1
	MFE23			88	11	10	1.5	6.7 : 1	4.3 : 1
	D29			87	4	5	1	5 : 1	7.6 : 1
7 (78)	Mock	6	Inoperable – extra-hepatic disease	99	0	0	0	—	1.9 : 1
	MFE23			99	23.9	15.6	7.9	2 : 1	1.8 : 1
	D29			99	2	1	0.3	3.3 : 1	1.8 : 1
8 (66)	Mock	4	Inoperable – extra-hepatic disease	99	0	0	0	—	8.8 : 1
	MFE23			99	19.9	15.5	1.9	8.1 : 1	7.0 : 1
	D29			99	2	1.3	0.2	6.5 : 1	8.4 : 1
9 (66)	Mock	24	Right hepatectomy	99	0	0	0	—	7.4 : 1
	MFE23			99	25	17	3	5.7 : 1	8.5 : 1
	D29			99	20	19	2	9.5 : 1	8.2 : 1
10 (57)	Mock	156	Left hepatectomy	99	2	0	0	—	4.2 : 1
	MFE23			99	14	18	3	6 : 1	4.0 : 1
	D29			99	9	10	1	10 : 1	4.6 : 1

All 10 patients recruited were due to undergo surgery for colorectal hepatic metastases. All the metastases removed or biopsed were confirmed as adenocarcinoma of colorectal origin by histological examination. T lymphocytes were isolated from these patients 2–3 weeks prior to surgery and were transduced with recombinant retroviruses encoding either MFE23.CD3ζTDGA or D29.CD3ζTDGA chimeric receptors. After expansion using an IL-2-based protocol, the T lymphocyte populations were analysed for the expression of the EGFP marker gene and for the surface markers CD3, CD4 and CD8 by staining with PE-conjugated antibodies and subsequent flow cytometric analysis using a FACscan (Becton Dickenson) with winMDI software.

.

### Patient T lymphocyte isolation and transduction

Approximately 20 ml of venous blood was obtained from each patient approximately 2–3 weeks prior to hepatic resection. Heparinised venous blood was diluted 1 : 1 ratio with serum-free RPMI 1640 media and mononuclear cells isolated by Ficoll-density gradient centrifugation (Ficoll–Histopaque (density 1077 g l^−1^) Sigma, Dorset, UK). Monocytes were depleted by adherence to plastic by culture for 1 h in tissue culture flasks at 37°C. Nonadherent lymphocytes were isolated and activated in 6-well nontissue culture plates precoated antibody (OKT-3 and anti-CD28) with 100 IU ml^−1^ interleukin-2 (IL-2) for at least 3 days. After activation, T lymphocytes were ficolled to remove dead cells, washed and counted. 1–4.5 × 10^6^ T lymphocytes were mixed with PG13-derived retroviral supernatant and polybrene (6 *μ*g ml^−1^) in a 50 ml falcon tube and centrifuged for 2–3 h at 1400 × **g**. Mock-transduced cells were mixed with T-cell media with polybrene instead of retroviral supernatant and centrifuged. After centrifugation, T lymphocytes were cultured overnight at 5 × 10^5^ ml with 100 IU ml^−1^ IL-2. Centrifugal transduction was repeated the next day and the resulting cell populations were maintained at 5 × 10^5^ cells ml^−1^ in 100 IU ml^−1^ IL-2. Fresh media and IL-2 were added every 2–3 days.

### Flow cytometry

Cells were washed with phosphate-buffered solution (PBS)/2% bovine serum albumin (BSA) and then resuspended in saturating concentrations of antibody (anti-CD3*ɛ*, CD4, CD8 and suitable isotype controls (all R-Phycoerythrin conjugated) purchased from Pharmingen, Oxford, UK) on ice for 30 min. Cells were then washed twice with PBS/2% BSA, fixed in 2% paraformaldehyde and analysed using a FACscan (Becton Dickenson, CA, USA) with winMDI version 2.8 analysis software.

### Cytotoxicity assays

Three days prior to cytotoxicity assay, T lymphocytes were cultured in low IL-2 (20 IU ml^−1^) to reduce background lymphokine-activated killer (LAK) activity (Gilham *et al*, 2002). An 8-h chromium release assay was performed as previously described (Gilham *et al*, 2002) with effector : target ratios from 50 : 1 to 6.25 : 1. Maximal release was obtained by coculture with 100 *μ*l 2% Triton X-100/PBS and spontaneous release by target cell culture in media alone. Percentage-specific lysis was determined by the following formula:


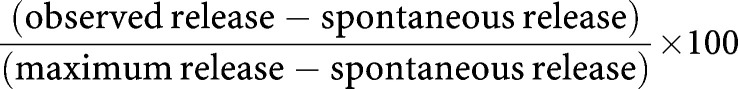


### Western blotting

Patient T lymphocytes were washed with PBS and lysed in RIPA buffer (150 mM NaCl, 1% NP-40, 0.5% deoxycholic acid, 0.5% sodium dodecyl sulphate (SDS) and 50 mM Tris-Cl pH 8.0) at the time of cytotoxicity assay and stored at −70°C. Approximately 5 × 10^5^ cells were separated by standard techniques using SDS/PAGE on 10% resolving gels (Lamelli, 1970). Hybond™ ECL™ nitrocellulose membrane (Amersham Pharmacia Biotech, Buckinghamshire, UK) was used for blots with anti-CD3*ζ* (clone 8D3) at 1 : 1000 dilution (Pharmingen, Oxford, UK) and a secondary of sheep anti-mouse HRP antibody at 1 : 2000 dilution (Sigma, Dorset, UK). Visualisation was performed using ECL™ reagents (Amersham Pharmacia Biotech, Buckinghamshire, UK).

### Tumour cell survival assay

Adherent tumour target cell lines (MKN45 K, LS174 T, LoVo and HeLa) were plated at a density of 5000 cells in 100 *μ*l of T cell media per well of flat-bottomed 96-well plates. These cells were allowed to adhere for 3 h at which point a further 100 *μ*l of T-cell media containing either varying numbers of transduced or control T lymphocytes or culture medium alone was added to the target cells to generate a final volume of 200 *μ*l. In some experiments, IL-2 was added to a final concentration of 100 IU ml^−1^. After a period of coculture, the supernatant containing nonadherent cells was removed and the remaining cells cultured in DMEM with 10% FBS without IL-2 for a further 5–6 days. The culture medium was aspirated, the cells washed once with PBS and 100 *μ*l of DMEM with 10% FBS containing a 40-fold dilution of Wst-1 reagent (Roche, Surrey, UK). At suitable time points, the optical density of the wells was determined using an ELISA plate reader (Molecular Devices, Sunnydale, CA, USA) set at 450–650 nm. Maximal cell growth was determined from the optical density reading of tumour cells cultured without T lymphocytes and minimal cell growth was determined from tumour cells cultured without T lymphocytes and lysed with 50 *μ*l of 2% Triton X-100/PBS. Relative tumour cell growth was estimated by the following equation:


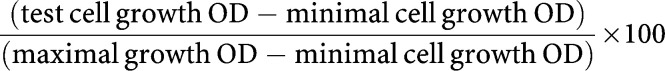


## RESULTS

### Transduction of patient T lymphocytes

Twenty millilitres of venous blood was obtained from patients 1 to 10 two to three weeks prior to the date of surgery. T lymphocytes were isolated and activated using immobilised anti-CD3 and anti-CD28 antibodies. After 3 days of antibody activation, 5–10 × 10^6^ viable T cells were counted by trypan blue exclusion for each patient indicating that in 10 out of 10 samples, T lymphocytes were successfully activated. Transduction with retroviruses encoding the anti-CEA CIR, MFE23.CD3*ζ*TDGA or the anti-NCAM CIR, D29.CD3*ζ*TDGA (Figure 1A) was performed using centrifugation in the presence of polybrene. After transduction, patient T lymphocytes were expanded in number by 5–10-fold over the next 7–21 days in the presence of 100 IU ml^−1^ of IL-2 (data not shown). Flow cytometry analysis confirmed the cell populations to consist largely of CD3-positive T lymphocytes (87–99%, Table 1). The IL-2-based expansion protocol supported the growth of the polyclonal T lymphocyte populations, although there was a relative predominance of CD8+ over CD4+ T cells. The lowest ratio of CD8 : CD4 was 1.5 : 1, while ratios in excess of 10 : 1 were recorded for two donors.

The level of retroviral transduction was estimated through the expression of the EGFP marker gene. A degree of heterogeneity was also observed with the levels of retroviral transduction varying between 2 and 33% (Table 1). In each patient sample, the ratio of transduced CD8 to transduced CD4 cells reflected the overall ratio of total CD8 to CD4 T cells in the total population suggesting there to be little bias in the susceptibility of either T-cell sub-type to transduction by the PG13-derived retroviruses.

Chimeric immune receptor expression in T lymphocytes was confirmed by Western blotting (Figure 1B). Nonreducing Western blots identified the formation of CIR homodimers and also heterodimers formed between the CIR and wild-type CD3*ζ*. Reducing Western blots demonstrated an immunoreactive band at the predicted molecular mass of the CIR (43 kDa) with the native CD3*ζ* protein present as a 16 kDa immunoreactive band.

### Specific cytotoxicity of CEA-positive cells by patient-modified T lymphocytes

**Figure 2:**
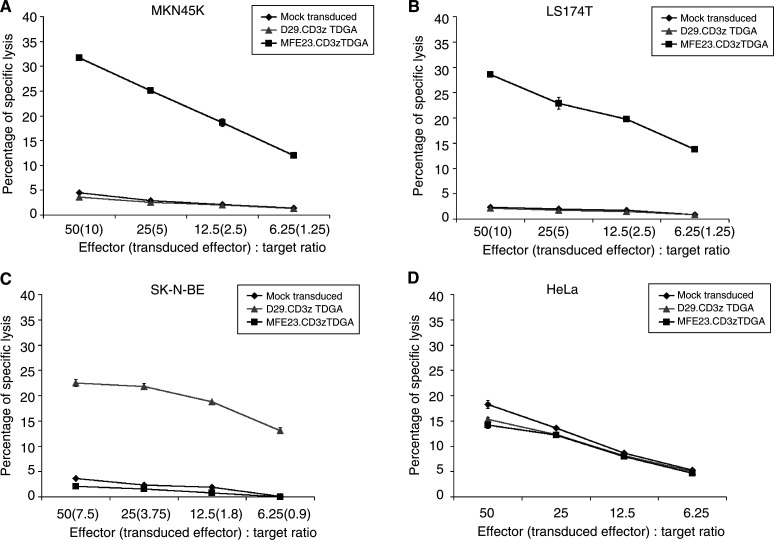
Chimeric receptor-expressing T lymphocyte populations demonstrate antigen-specific killing of tumour cell lines. T lymphocytes derived from Patient 1 expressing either the MFE23.CD3*ζ*TDGA (▪) or the D29.CD3*ζ*TDGA (▴) chimeric receptors or control mock-transduced T cells (♦) were cocultured with 5000 ^51^Cr-labelled target cells per well at the indicated effector : target ratios. (The transduced effector : target ratio based upon EGFP fluorescence is also presented.) After 8 h, 100 *μ*l of media was harvested and analysed for released ^51^Cr label (Topcount, Packard, Berkshire, UK). Maximal lysis was determined by lysis of target cells by 2% Triton X-100/PBS. Target cell lines used were MKN45 K (**A**), LS174 T (**B**), SK-N-BE (**C**) and HeLa (**D**). The results presented are the mean±s.d. of triplicate wells.

MFE23.CD3*ζ*TDGA-modified lymphocytes from patient 1 demonstrated specific cytotoxic activity against the CEA-expressing gastric tumour cell line MKN45 K and the colon adenocarcinoma LS174T cell line. Negligible cytotoxicity was observed when NCAM-targeted (D29.CD3*ζ*TDGA) and mock-transduced T lymphocytes were cocultured with the MKN45 K or LS174 T cell lines (Figures 2A and B

). Specific CEA-targeted lysis was found to be increased by at least seven-fold over that of nontargeted lymphocytes.

The specificity of cell killing was confirmed by the cytotoxic activity of D29.CD3*ζ*TDGA-transduced T lymphocytes against NCAM-positive neuroblastoma SK-N-BE target cells (Figure 2C). In this case, low background activity was found when CEA-targeted (MFE23.CD3*ζ*TDGA) and mock-transduced T lymphocytes were cultured with CEA-negative SK-N-BE cells (Figure 2C). HeLa cells (NCAM- and CEA-negative) were used to demonstrate equivalent nonspecific killing by all three populations thereby confirming that the specific killing observed was because of CIR activity and not because of any altered killing properties of the individual lymphocyte populations (Figure 2D). The level of retroviral transduction (as assessed by the expression of EGFP) was 20% in the MFE23.CD3ζTDGA CIR-expressing population generated from patient 1. This figure indicates that the maximal transduced effector to target ratio was 10 : 1 with highly specific cytolytic activity observed down to a ratio of 1.25 transduced effector cells per target (Figure 2A). In the case of NCAM-targeted cells (SK-N-BE, Figure 2C), the proportion of D29.CD3*ζ*TDGA-expressing cells was estimated to be 15%, hence, the transduced effector to target ratio was maximally 7.5 : 1 with clear specific cytolytic activity at the lowest ratio tested (0.9 transduced T cells per target cell).

**Figure 3:**
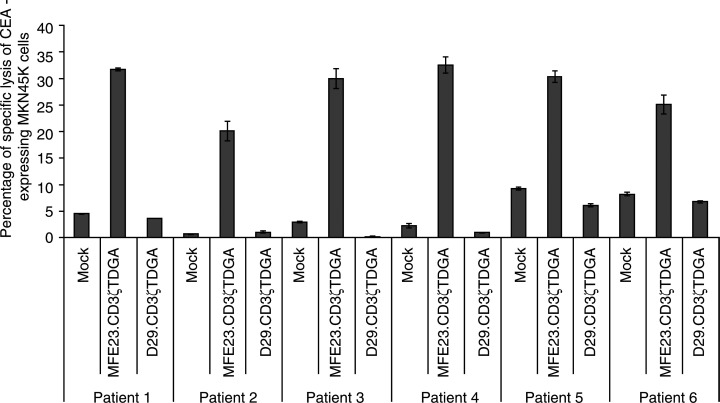
Antigen-specific cytotoxicity mediated by chimeric receptor expressing T lymphocytes generated from five patients with advanced colorectal cancer. A summary of the cytotoxic activity of T-cell populations generated from Patients 1 to 6 expressing either the MFE23.CD3*ζ*TDGA or the D29.CD3*ζ*TDGA chimeric receptors or control, mock-transduced T cells. Standard 8-h chromium release assay was performed using MKN45 K gastric carcinoma cells as targets with the T-cell effectors at an effector : target ratio of 50 : 1. The results presented are the mean±s.d. of triplicate wells.

Specific, targeted cytotoxicity was subsequently demonstrated for the remaining five patient lymphocyte samples against MKN45 K gastric carcinoma cells (Figure 3

). In each case, specific, dose-dependent killing was observed. Furthermore, in each case, very low background levels of killing were observed (D29.CD3*ζ*TDGA and mock-transduced lymphocytes). This probably reflects the period of preculture in low concentrations of IL-2 reducing nonspecific cytokine-mediated killing activity. Equivalent specific lysis was observed between the various transduced cell populations when tested against CEA-negative, NCAM-negative target cells indicating there to be no difference in the overall killing activity of the modified or nonmodified T lymphocyte populations (data not shown).

A consideration of the level of retroviral transduction (as assessed by EGFP expression) did not appear to provide a clear relation between the levels of expression of the CIRs with the observed CIR-mediated cytotoxic activities. There was maximally a 6.6-fold difference between these six patient samples in retroviral transduction frequency, yet the cytotoxic activity varied only 1.6-fold. It was clear that the lowest level of transduction correlated with the weakest cytolytic activity (Patient 2; 5% CD3+, GFP+; 20.1% specific killing). However, there appeared little difference in cytolytic activity in these populations once a transduction level of 20% had been achieved.

### Tumour cell survival after coculture with gene-modified T lymphocytes

Short-term assessments of cytolytic activity clearly demonstrated that CIR-expressing T-cell populations were functional against protein antigen targets in an MHC-independent manner. In an attempt to investigate and understand the functionality of these gene-modified cell populations further, and in an effort to quantify the overall efficiency of the targeted gene modified cells, longer-term tumour cell survival assays were used to assess the effectiveness of these T cells. Adherent tumour cell lines were cocultured with nonadherent T lymphocytes for set periods of time, after which the nonadherent cells were removed and the tumour cells cultured in media without cytokines to ensure that any remaining T cells would not survive. Five to six days, later, the growth of the tumour cells was assessed using an MTT-based assay (Mosmann, 1983).

**Figure 4:**
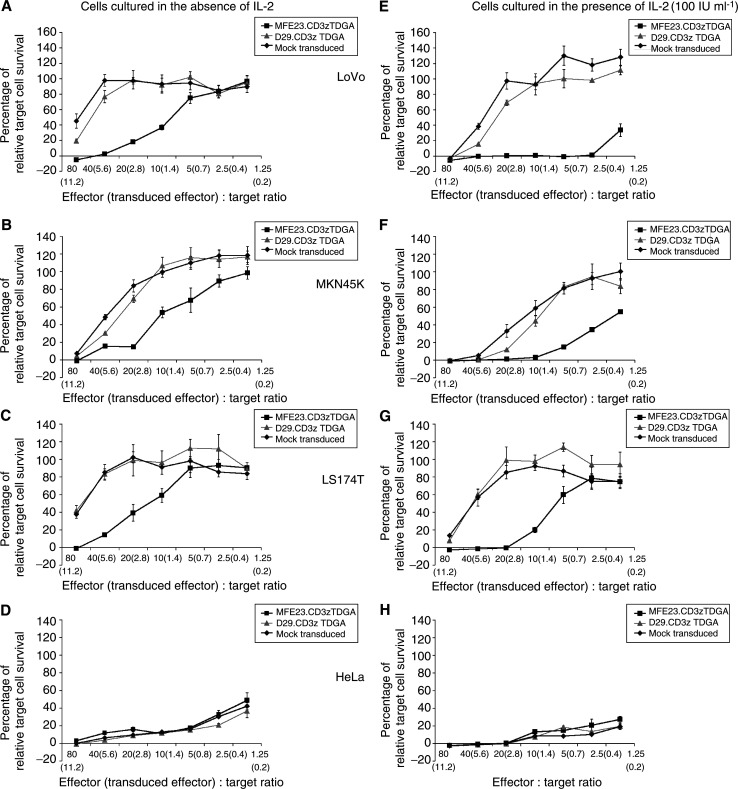
Tumour target cell survival after coculture with targeted and nontargeted T lymphocytes. Adherent tumour cell lines (5000) were cultured with either MFE23.CD3*ζ*TDGA (▪) or the D29.CD3*ζ*TDGA (▴) chimeric receptors or control mock-transduced T cells (♦), generated from Patient 10, at the indicated effector : target ratios (The transduced effector : target ratio based upon EGFP fluorescence is also presented.) in wells of flat-bottomed 96-well plates in a final volume of 200 *μ*l. Interleukin-2 was added to some wells at a final concentration of 100 IU ml^−1^ (E–H). After 24 h, media and nonadherent cells were removed and replaced with target cell culture medium without T-cell cytokines. After a further 5 days of culture, the wells were aspirated, washed with PBS and 100 *μ*l of DMEM/10% FBS containing a 1 : 40 dilution of wst-1 reagent (Roche, Surrey, UK). The optical density of the wells was assessed at 450–650 nm using an ELISA plate reader (Molecular Devices, Sunnydale, CA, USA). Maximal tumour cell growth was assessed from the optical density of wells containing tumour cells only and minimal growth determined from wells containing tumour cells lysed with 2% Triton X-100. Target cell lines used were MKN45 K (**A**), LS174 T (**B**), SK-N-BE (**C**) and HeLa (**D**). The results are presented as the mean±s.d. values of quadruplicate wells.

Multiple cell lines were used in order to examine the activity of the anti-CEA-targeted T cells. The CEA-expressing human colon adenocarcinoma lines (LS174 T and LoVo) as well as the gastric carcinoma cell line (MKN45 K) were tested as targets along with HeLa cells used as a control since they do not express CEA. This subsequent work utilised T lymphocytes generated from four further patients (7–10, Table 1). The results depicted in Figure 4

represent the activity of cells generated from Patient 10 and are representative of experiments performed with Donors 7–10.

The first observation was that the cell lines used displayed a differential degree of susceptibility to coculture with transduced or control T lymphocytes. HeLa cells proved to be the most sensitive in that very low levels of target cell survival were recorded when cocultured for 24 h in the presence of T cells at an effector : target ratio approaching 1 : 1 (Figure 4D). However, it was clear that there existed no difference in the sensitivity of HeLa cells to either transduced or nontransduced T cells, indicating that there was equivalent nonspecific killing activity between the T-cell populations in agreement with the results observed in chromium release assay. The specific effect of T-cell targeting was observed in assays using CEA-expressing tumour cell lines. In each case, tumour cell survival was reduced in a dose-dependent manner when the target cells were cocultured with MFE23.CD3*ζ*TDGA T-cell populations as compared to either NCAM-targeted or control nontransduced T cells (Figure 4A–C). There was a clear difference in target cell growth observable at effector : target ratios of 10 : 1, which represented a transduced effector : target ratio of 1.4 : 1. However, only at the highest cell dose (80 : 1 total cells, 11.2 : 1 transduced effector:target ratio) there was no tumour cell growth observed by all three target cell lines after a 24-h period of coculture. The susceptibility of the gastric (MKN45 K) and adenocarcinoma (LoVo and LS174 T) cell lines to nonspecific killing was reduced compared to that of HeLa cells. However, in the case of LoVo and LS174 T cells, the highest dose of nontargeted control T cells resulted in a reduction in target cell survival to levels approximately 40% of maximal (Figures 4A and C). For MKN45 K target cells, there appeared to be an equivalent degree of antitumour activity in targeted (MFE23.CD3*ζ*TDGA) and nontargeted (D29.CD3*ζ*TDGA and mock-transduced T cells) at the maximum dose of effector cells (Figure 4B). Microscopic examination of the culture wells confirmed that large adherent tumour cells were present in the wells identified as having metabolic activity within the assay with few lymphocytic cells present. This indicated that attached lymphocytes made a minimal contribution to the metabolic activity of the wells in agreement to similar assays performed elsewhere (Hombach *et al*, 2001).

The inclusion of IL-2 during the period of coculture drastically enhanced the effectiveness of target cell killing. For control HeLa cell targets, a maximal repression of target cell growth was observed at an effector : target ratio of 20 : 1 compared to a ratio of 80 : 1 required in the absence of this cytokine. Once again, there was little difference between the effectiveness of each T-cell population in repressing HeLa cell growth (Figure 4H). A similar degree of enhanced antitumour activity was recorded after coculture of CEA-expressing cell lines with the T-cell populations. Maximal tumour cell growth repression was found at lower doses of effector cells than in the absence of IL-2. Total repression was found at effector : target ratios of 20 : 1 (2.8 transduced MFE23.CD3*ζ*TDGA cells : 1 target) for both LS174 T and MKN45 K cells, while the low dose of 2.5 : 1 (0.4 transduced cells per effector target) resulted in maximal LoVo cell killing. The specificity of activity was maintained with a clear difference in the cytolytic activity of targeted and nontargeted (NCAM and mock-transduced cells) being evident (Figures 4E–G). However, enhanced nonspecific, cytokine-driven killing activity was observed against each cell line since a total repression of tumour cell growth occurred at lower effector doses than that observed in the absence of IL-2.

## DISCUSSION

Recent studies have shown that T lymphocytes can be redirected against cancer cell lines using CEA as a target antigen through the use of CIRs expressed in T cells isolated from healthy donors (Nolan *et al*, 1999; Beecham *et al*, 2000; Daly *et al*, 2000; Hombach *et al*, 2000). In our study, the chimeric receptor consisted of an extracellular antigen-binding domain (scFv) specific for CEA (Chester *et al*, 1994; Begent *et al*, 1996), attached to the CD3*ζ* component of the T-cell receptor complex. This CIR has already been demonstrated to be effective in initiating cytokine secretion and the cytotoxic activities of normal donor T lymphocytes (Gilham *et al*, 2002).

In the light of published work demonstrating the dysfunctional properties of T lymphocytes isolated from patients with a cancer load (Rosenberg *et al*, 1994; Bukowski *et al*, 1998; Yoong and Adams, 1998), the aims of this project were to assess whether the conditions used to activate and transduce T lymphocytes from normal donors were equally effective in T lymphocytes from patients with advanced colorectal cancer. In addition, it is important to evaluate whether CIR crosslinking in the presence of antigen would generate a response in patient cells similar to that of normal donors.

Ten patients with advanced colorectal cancer were recruited into this study. T lymphocytes were successfully transduced with efficiencies ranging from 2 to 33% (mean 15±9%, *n*=20) for all viruses and from 5 to 33% (mean 19.4±7.8%) for MFE23.CD3*ζ*TDGA-transduced T cells (Table 1). It is clear that higher levels of transduction are preferable particularly with respect to use in a clinical context. Protocols have been devel-oped that have reported high-level gene transfer using longer antibody-mediated preactivation periods (Movassagh *et al*, 2000), through the use of retronectin to enhance infection (Hanenberg *et al*, 1996) or by preloading plates with retrovirus (Kuhlcke *et al*, 2002). The levels of retroviral transduction generated here represent those achieved using a short period of centrifugation (spin-fection) and is adaptable to transduction on a large scale. An over-riding issue concerns the development of such protocols into a clinically applicable process using good manufacturing process (GMP) guidelines. Clinical trials using chimeric receptor expressing T lymphocytes against HIV (Mitsuyasu *et al*, 2000) and current trials using plasmid-transfected T cells expressing chimeric receptors (Jensen *et al*, 2000) have demonstrated the feasibility of this approach, and efficient gene-transfer protocols are now being developed based upon this experience. As a result, the optimal protocol may or may not feature some of the methods described above. As such, the use of a spin-fection protocol permitted levels of transduction that were suitable to permit the analysis of CIR activity in primary patient T cells.

After transduction, all 10 of the patient T-cell samples were expanded using an IL-2-based expansion protocols. The level of expansion reported here using IL-2 alone ranged from five- to 10-fold above the starting number. For clinical application, an ideal scenario would involve the generation of large numbers of T cells from, for example, a single blood collection or leucophoresis. It would be anticipated that a larger scale expansion of patient-derived lymphocytes would be required. Preliminary studies have shown that the inclusion of soluble antibodies (OKT3 and anti-CD28) with IL-2 enhanced the level of expansion above that with IL-2 alone (data not shown). These experiments have used standard media with FBS and, as such, do not represent conditions proposed for use in a clinical situation. Our efforts are now directed to investigating the growth of patient-derived T lymphocytes using GMP conditions reported for the maximal growth of T lymphocytes elsewhere (Carlens *et al*, 2000). However, the fact that all 10 patient samples were successfully transduced and expanded was encouraging, considering the reported frailties of patient-derived T lymphocytes (Mizoguchi *et al*, 1992; Rosenberg *et al*, 1994; Bukowski *et al*, 1998).

The CIR-bearing cell populations responded to antigen present on cell lines through specific target cell lysis in an MHC-independent manner. Importantly, even in the T-cell population with the lowest transduction level, significant antigen-specific cytotoxic activity was observed. Interestingly, increasing the level of retroviral transduction did not result in an increasing functional cytotoxic activity. Once a transduction level of 20% had been achieved, there appeared to be little increase in overall antigen-specific cytotoxicity as assessed by chromium release assay. These observations, albeit on a restricted number of transduced patient samples, indicates that more complicated factors may be at work. Further work is required to determine whether the killing activity generated by the transduced population is mediated by a few modified cells acting with high efficiency or whether the majority of CIR-expressing cells are acting at lower efficiency in order to generate the antigen-specific cytotoxicity observed here. This is important because further development of this approach may require optimisation of the receptor in order to produce a stronger response in cells where killing efficiency is low, while a modification to T-cell activation and culture conditions may be required to generate a greater number of highly cytotoxic T cells.

Interestingly, the specific production of interferon gamma from the T-cell cultures in the presence of antigen was less clearcut. Although there was evidence of specific cytokine production from some donors, in others there was either high background or poor levels of overall cytokine secretion (data not shown). The reasons for this are unclear, but were not related to cytotoxic activity.

An alternative to the chromium release assay was used to further investigate the potential of the gene-modified T lymphocytes. Tumour cell survival was assessed after a period of coculture with T cells. These assays potentially assess the longer-term interactions of effector and target cells, which may affect target cell survival through processes such as the induction of apoptosis through cytokine activity or death receptor activation.

An important observation was the fact that there was a difference in the overall sensitivity of the individual tumour cell lines to coculture with allogeneic T cells. This feature may be reflected *in vivo* where tumours would most likely show a variation in susceptibility to T lymphocyte activity. HeLa cells proved to be most sensitive to nonspecific killing in the tumour cell survival assay. In comparison, the colon adenocarcinoma and gastric cell lines were more resistant to nonspecific killing, although these cell lines were efficiently targeted by anti-CEA CIR-expressing T-cell populations. Coculture in the presence of IL-2 drastically enhanced both the nonspecific and specific killing activities of the T-cell populations. A total repression of target cell growth was seen in effector : target ratios as low as 2.8 transduced T cells per target for MKN45 K and LS174 T cells and as low as 0.4 transduced cells per target for LoVo cells. It is not clear whether the enhanced cytolytic activity is through enhanced lymphokine-activated killer (LAK) activity of the T cells resulting in the T cells being more active in cytolytic assays or whether the IL-2 was acting as a survival factor effectively enhancing the survival of the T cells (which had previously been expanded in IL-2), thereby permitting an enhanced killing activity. In either case, it is clear that cytokine help is potentially of crucial importance in the clinical setting through the reduction of the number of T cells required to target a tumour mass.

From the *in vitro* data presented here, the addition of IL-2 resulted in at least a four-fold decrease in the cell number required to effect a total repression of tumour cell line growth. It is impossible to apply *in vitro* assay results directly to the problem of dealing with tumour masses *in vivo*. However, this work suggests that for CIR-expressing T lymphocytes to function in a practical form *in vivo*, the CIR-modified cells will require some help in the form of survival or activation signals. These signals may take the form of infusions of IL-2. However, IL-2 administration is associated with toxicity. Alternative studies in our own group have investigated the ability of antiapoptotic genes to act as survival factors which could be expressed from a single retroviral construct (Eaton *et al*, 2002). Recent developments in CIRs have also involved the generation of receptors composed of the CD3*ζ* domain fused to the CD28 receptor signalling chain (Hombach *et al*, 2001; Maher *et al*, 2002). These fusion receptors generate both a cytotoxic response and a costimulatory signal (and therefore IL-2 production) in an attempt to more closely mimic the events that naturally occur when T cells encounter antigen-producing cells in order to produce an effective immune response.

Our data indicate that T lymphocytes derived from patients with advanced colorectal cancer are able to mount effective, MHC-independent, functional responses after transduction with CIR-encoding recombinant retroviruses. Other studies have shown that T lymphocytes isolated from patients with ovarian (Parker *et al*, 2000) and prostate (Gong *et al*, 1999) cancers are also susceptible to retroviral transduction and CIR-mediated functional activity. These studies together demonstrate that patient-derived T lymphocytes are indeed suitable as vehicles for this type of gene modification and that CIR technology is highly flexible through the use of antibody recognition enabling virtually any protein antigen to be targeted by T lymphocytes. However, it is also clear from this work that a further understanding of CD3*ζ*-based CIR activities and aspects of manipulated T lymphocyte biology, particularly with respect to their function *in vivo* and the development of practical GMP-based handling methods, are required to ensure that this approach is fully adapted for clinical trial.
